# Antibody selection and automated quantification of TRPV1 immunofluorescence on human skin

**DOI:** 10.1038/s41598-024-79271-9

**Published:** 2024-11-18

**Authors:** Yuying Jin, Julian Brennecke, Annemarie Sodmann, Robert Blum, Claudia Sommer

**Affiliations:** Department of Neurology, University Hospital of Würzburg, 97080 Würzburg, Germany

**Keywords:** Transient receptor potential vanilloid 1 (TRPV1), Immunofluorescence, Deep learning, Machine learning, Bioimage analysis, Computational neuroscience, Peripheral nervous system

## Abstract

Assessing localization of the transient receptor potential vanilloid-1 (TRPV1) in skin nerve fibers is crucial for understanding its role in peripheral neuropathy and pain. However, information on the specificity and sensitivity of TRPV1 antibodies used for immunofluorescence (IF) on human skin is currently lacking. To find a reliable TRPV1 antibody and IF protocol, we explored antibody candidates from different manufacturers, used rat DRG sections and human skin samples for screening and human TRPV1-expressing HEK293 cells for further validation. Final specificity assessment was done on human skin samples. Additionally, we developed two automated image analysis methods: a Python-based deep-learning approach and a Fiji-based machine-learning approach. These methods involve training a model or classifier for nerve fibers based on pre-annotations and utilize a nerve fiber mask to filter and count TRPV1 immunoreactive puncta and TRPV1 fluorescence intensity on nerve fibers. Both automated analysis methods effectively distinguished TRPV1 signals on nerve fibers from those in keratinocytes, demonstrating high reliability as evidenced by excellent intraclass correlation coefficient (ICC) values exceeding 0.75. This method holds the potential to uncover alterations in TRPV1 associated with neuropathic pain conditions, using a minimally invasive approach.

**Supplementary Information:**

The online version contains supplementary material available at 10.1038/s41598-024-79271-9.

## Introduction

The transient receptor potential vanilloid-1 (TRPV1) ion channel is a protein expressed on primary afferent neurons, where it responds to various stimuli such as heat, low pH, and inflammatory mediators^1,2^. Its activation plays a crucial role in detecting nociceptive signals and facilitating the body’s response to harmful stimuli^3^. The clinical relevance of TRPV1 in pain is exemplified by the profound changes in nociception in people with a missense mutation of the TRPV1 gene^4^. Other TRPV1 channel variants are associated with more severe pain, as in diabetic or small fiber neuropathy^5,6^. This suggests a complex relationship between TRPV1 variants and pain perception. Small molecule TRPV1 antagonists are still in development as novel analgesics^7^. Topical treatment with high-dose capsaicin, a TRPV1 agonist, is an effective therapy for focal neuropathic pain^8^ and works by temporarily silencing TRPV1-expressing nerve fibers in the skin. In addition to its role in pain modulation^9^, TRPV1 signaling has also been implicated in skin aging, and it conveys anti-inflammatory and potential anti-cancer effects^10^. Notably, TRPV1 is not only present in axons but also in keratinocytes and immune cells^10,11^. Therefore, assessing TRPV1 localization in skin accurately may be challenging.

TRPV1 in skin might be a biomarker for early-stage disease detection and could be developed into a target for therapeutic interventions. Skin biopsy serves as a minimally invasive tool for investigating neuropathies, with intra-epidermal nerve fiber density providing a valuable parameter in the diagnosis of small fiber neuropathy^12^. The examination of cutaneous sensory neurons expressing nociceptive receptors, including TRPV1, is pivotal for our understanding of pain transmission. The detection of changes in the expression and spatial aspect of TRPV1 through immunofluorescence (IF) may unveil mechanisms underlying neuropathies and might become an important addition to standard skin histology.

Although TRPV1 antibodies designed for human tissue are available from various manufacturers, the reliability of these antibodies is not well-established. Additionally, while IF staining methods for TRPV1 have been applied in rodent skin biopsies^13^, a standardized protocol for human skin biopsy is currently lacking.

Detection of TRPV1 in peripheral nerve fibers often involves multi-color IF. Analysis of the microscopy bioimages frequently relies on the individual detection criteria of the expert^14^. To ensure improved objectivity in bioimage analysis, strategies based on machine-learning or deep-learning have been established^15,16^.

In this study, we have developed a reliable TRPV1 IF staining protocol for human skin biopsies. Furthermore, we experimentally tested and implemented objective quantification of TRPV1 abundance in nerve fibers of the skin.

The methods described here can be used to advance our understanding of TRPV1 expression in human skin and will potentially help to uncover nociceptive mechanisms and guide therapy.

## Results

### Antibody performance on rat dorsal root ganglion (DRG) and human skin

Given the reasonable correlation (*r* = 0.81) between protein expression profiles in rat and human DRGs^17^, we conducted a preliminary screening of TRPV1 antibody candidates on rat DRG sections, a suitable model to test natural TRPV1 expression. Human skin sections, containing both nerve fibers and keratinocytes that express TRPV1, were used in parallel.

**Fig. 1:**
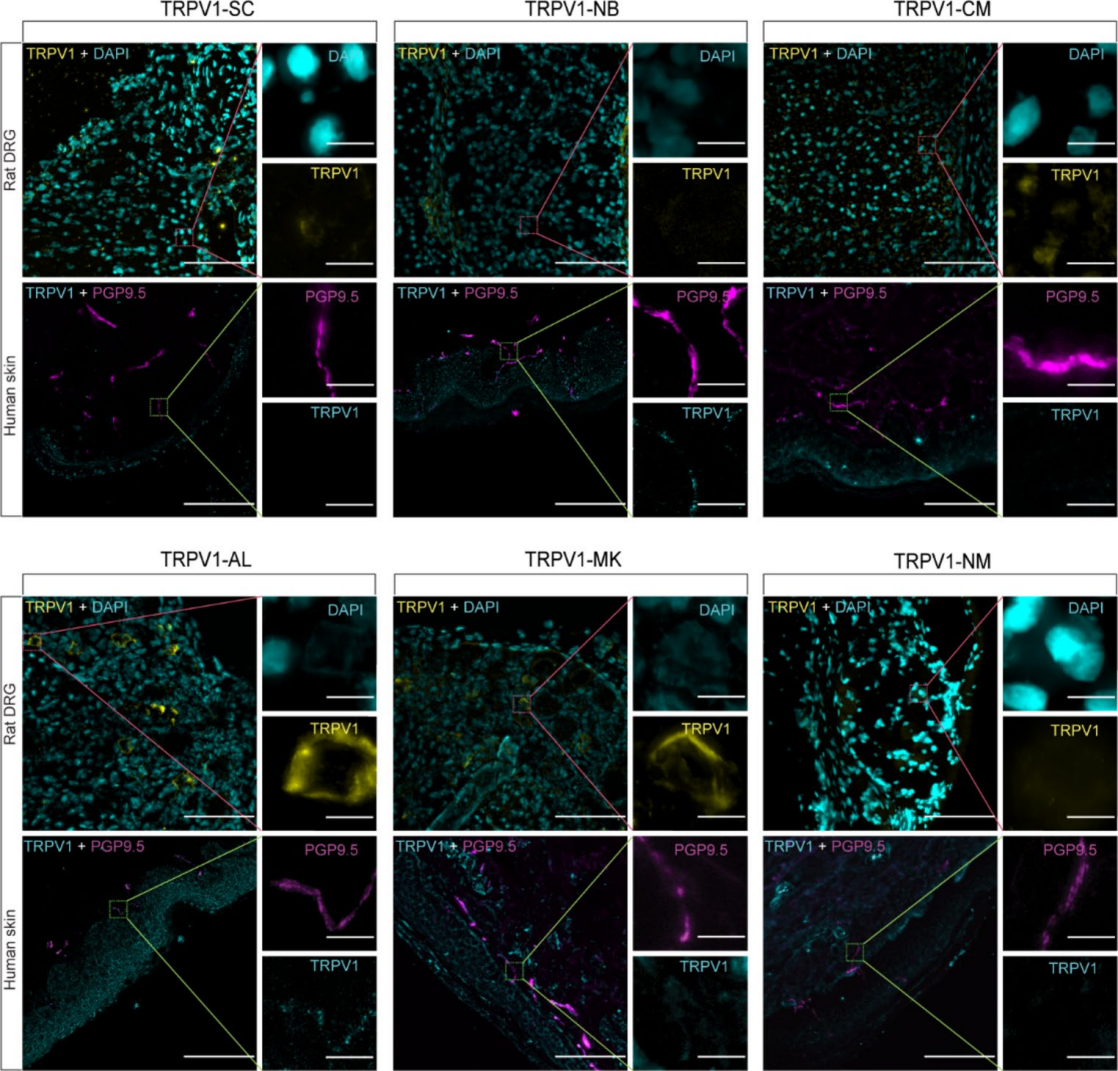
Assessment of TRPV1 antibodies using rat DRG and human skin. IF labeling was performed using various anti-TRPV1 antibodies on rat DRG and human skin sections, with corresponding antibodies indicated. Notably, TRPV1-NB and TRPV1-AL show immunoreactivity on nerve fibers in human skin. Scale bar = 100 μm–5 μm (for the zoomed-in views).

According to our IF staining results (Fig. 1), TRPV1-AL and TRPV1-MK antibodies showed obvious TRPV1 signals on rat DRG sections. TRPV1-SC, TRPV1-CM and TRPV1-NM antibodies showed unsatisfactory performance on the rat DRG, with only weak immunoreactive signals. The TRPV1-NB antibody reacted solely to human samples, but not on rat DRG.

In human skin sections, TRPV1-SC, TRPV1-CM, TRPV1-MK, and TRPV1-NM antibodies did neither label keratinocytes in the epidermis nor nerve fibers, indicating a lack of sensitivity. In contrast, exclusive labeling of distinct immunoreactive puncta on both the keratinocytes and nerve fibers was observed with TRPV1-NB and TRPV1-AL antibodies.

### Specificity of TRPV1 antibodies on transfected HEK293 cells

**Fig. 2:**
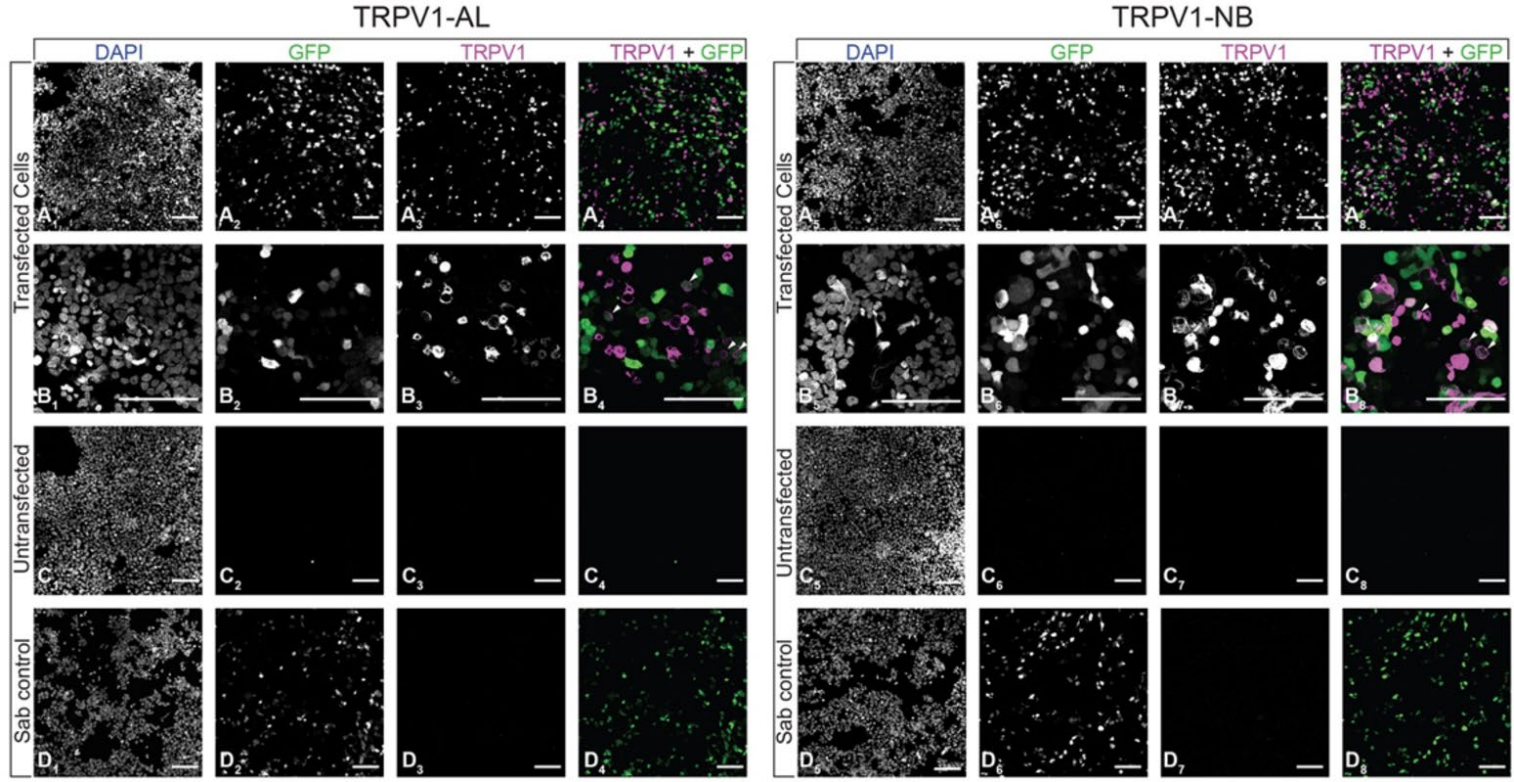
Testing anti-TRPV1 antibodies on hTRPV1-expressing HEK293 cells. TRPV1-AL (left) and TRPV1-NB (right) were used to label HEK293 cells expressing human TRPV1. GFP was expressed from a second vector and was used as a transfection control. Experimental conditions are indicated on the y-axis. Row B shows a larger magnification of cells from A. Sab control: secondary antibody control. Scale bar = 100 μm.

To test the reliability of our findings, we conducted a sensitivity and specificity analysis, focusing on signal detection in HEK293 cells overexpressing recombinant human-TRPV1 (hTRPV1). GFP was expressed from a second vector and was used as a transfection control. Based on an adequate number of cells (Fig. 2A_1_, A_5_, B_1_, B_5_, C_1_, C_5_, D_1_, D_5_), a positive GFP signal indicates successful transfection (Fig. 2A_2_, A_6_, B_2_, B_6_, D_2_, D_6_), while the absence of a GFP signal serves as negative controls (Fig. 2C_2_, C_6_). Row B (Fig. 2B_1_–B_8_) provides a zoomed-in version of row A (Fig. 2A_1_–A_8_). The merged images for GFP and TRPV1 are shown in the last column for each (Fig. 2A_4_, A_8_, B_4_, B_8_, C_4_, C_8_, D_4_, D_8_).

In this test, both TRPV1-AL and TRPV1-NB antibodies exhibited distinct labeling of cells expressing hTRPV1 (Fig. 2B_3_, B_7_). Untransfected HEK293 cells were not labeled by these antibodies (Fig. 2C_3_, C_7_). Transfected cells stained with a single secondary antibody were only labeled by GFP (Fig. 2D_2_, D_6_) and not by the TRPV1 antibody (Fig. 2D_3_, D_7_), thereby excluding secondary antibody interference. This shows that both TRPV1-AL and TRPV1-NB antibodies effectively detected cells expressing hTRPV1, demonstrating specificity.

### TRPV1 antibodies in human skin with and without pre-adsorption with a blocking peptide

**Fig. 3:**
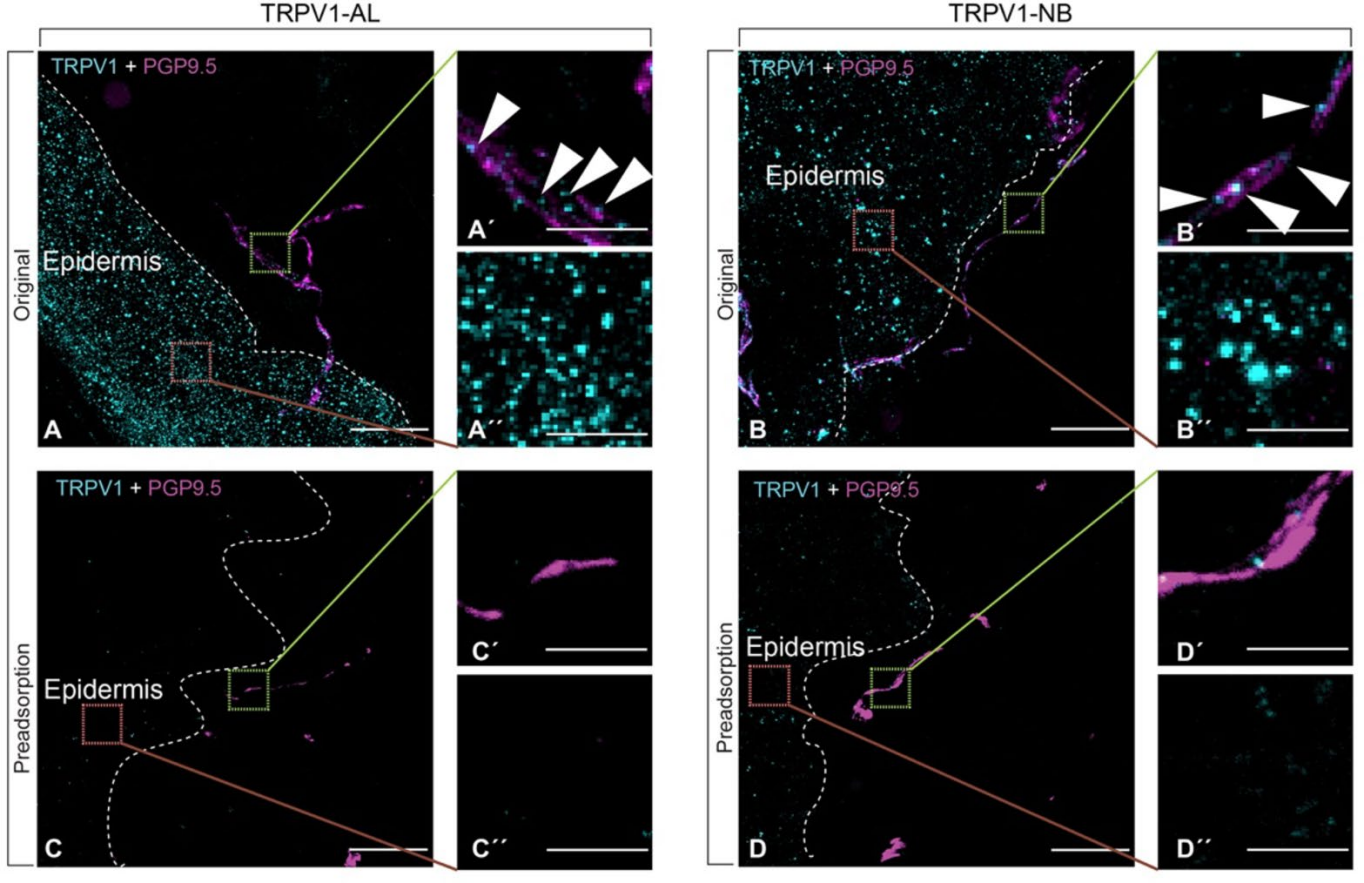
Immunofluorescence labeling of TRPV1 in human skin. PGP9.5-positive nerve fibers are shown in magenta, hTRPV1 labels are shown in cyan. The epidermis is indicated. The upper panels (**A**,**B**) show original TRPV1 labels. Images in (**A**′,**B**′) provide detailed views of TRPV1 IF on nerve fibers in the sub-epidermal region, while (**A**ʺ,**B**ʺ) show TRPV1 IF mostly in keratinocytes in the epidermis. The lower panels (**C**,**D**) present the loss of anti-TRPV1 immunoreactivity after application of blocking peptides against anti-TRPV1 (preadsorption). Scale bars = 20 μm–5 μm (for the zoomed-in views).

Given that the TRPV1-AL and TRPV1-NB antibodies showed similar performance on transfected HEK293 cells, we further tested their specificity in human skin. In the absence of antigen adsorption, both antibodies (TRPV1-NB and TRPV1-AL) exhibited distinct and bright, round immune signal puncta on keratinocytes in the epidermis (Fig. 3Aʺ, Bʺ) and on nerve fibers (Fig. 3A′, B′). This observation served as a robust positive control for subsequent antigen pre-adsorption tests (Fig. 3A, B).

After pre-adsorption with the corresponding TRPV1 antigen (blocking peptide) (Fig. 3C, D), the TRPV1-AL antibody was no longer able to generate immunoreactive puncta on nerve fibers and epidermis (Fig. 3C′, Cʺ). Similarly, with TRPV1-NB, immunoreactivity on nerve fibers and epidermis was significantly diminished by peptide pre-adsorption (Fig. 3D′, Dʺ).

### Overall results for immunostaining performance

The experimental results, as presented in Table 1, revealed variations in sensitivities and specificities among different antibodies across tissues. The TRPV1-MK antibody exhibited sensitivity on rat DRG but did not detect TRPV1 signals in human skin. In contrast, the TRPV1-AL antibody stained both rat DRG and human skin, with robust sensitivity and high specificity on TRPV1-transfected HEK293 cells, positioning it as a promising candidate for further investigation. Additionally, the TRPV1-NB antibody also performed well, exclusively reacting with human tissues and displaying positive sensitivity on human skin and hTRPV1-transfected HEK293 cells. In summary, TRPV1-AL and TRPV1-NB antibodies emerged as promising candidates for further studies based on their favorable performance in sensitivity and specificity assessments across different tissues.

**Table 1:** Specificity and sensitivity results of TRPV1 antibodies.

Abbreviation	Sensitivity on rat DRG	Sensitivity on human skin	Sensitivity on transfected HEK293 cells	Specificity on transfected HEK293 cells	Labeling of human skin
TRPV1-SC	+	−	Excluded	Excluded	Excluded
TRPV1-CM	+	−	Excluded	Excluded	Excluded
TRPV1-AL	++	+	+	+	+
TRPV1-MK	++	−	Excluded	Excluded	Excluded
TRPV1-NM	+	−	Excluded	Excluded	Excluded
TRPV1-NB	only human	+	+	+	+

### Comparison of methods for quantifying TRPV1 immunoreactive characteristics

**Fig. 4:**
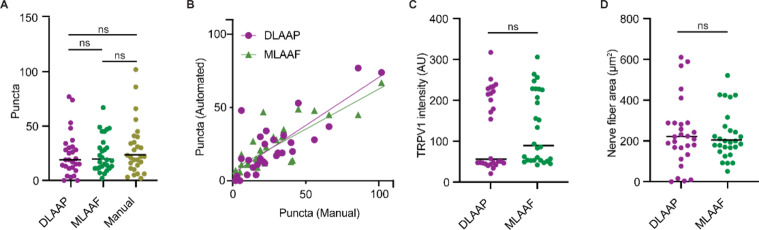
Comparison of TRPV1 quantification methods in human skin. (**A**) The number of TRPV1-immunoreactive puncta (y-axis) was quantified using three different bioimage-analysis strategies: Deep-learning-based automated analysis (DLAAP, purple), machine-learning-based automated analysis (MLAAF, green), and manual counting (yellow). The mean values for each method are indicated. Statistical analysis is provided in Table 3. (**B**) The TRPV1 punctum counts (y-axis) by DLAAP (purple) and MLAAF (green) correlate with the results from manual counting (r²_MLAAF−manual_ = 0.770, *p* < 0.001; r²_DLAAP−manual_ = 0.751, *p* < 0.001). (**C**) The anti-TRPV1-mediated mean fluorescence intensity (in AU, arbitrary units) on nerve fibers was measured using DLAAP (purple) and MLAAF (green). (**D**) The nerve fiber area (y-axis) was computed by DLAAP (purple) and MLAAF (green). ns = not significant.

TRPV1 immunoreactive puncta located on nerve fibers in bioimages were quantified using three different methods: machine-learning-based automated analysis in Fiji (MLAAF), deep-learning-based automated image analysis in Python (DLAAP), and heuristic manual annotation (Fig. 4; Table 2). When we compared the number of TRPV1 + puncta (Fig. 4A), the correlation between number of automatically annotated puncta and manually annotated puncta (Fig. 4B), mean signal intensity values of annotated TRPV1 + puncta (Fig. 4C), and annotated nerve fibers areas (Fig. 4D), no significant difference between analysis strategies was found. Moreover, a strong positive linear correlation was identified in the counts of TRPV1 immunoreactive puncta between MLAAF, DLAAP, and manual analysis (r²_MLAAF−manual_ = 0.770, *p* < 0.001; r²_DLAAP−manual_ = 0.751, *p* < 0.001) (Table 3; Fig. 4B).

We also tested the reliability of automated analysis methods using the intraclass correlation coefficient (ICC) calculation. For this, we compared MLAAF and DLAAP for the TRPV1 puncta quantification, signal intensity (mean fluorescence intensity; MFI), and nerve fiber area (Table 4; Fig. 4C, D). According to this statistical analysis, high ICC values (greater than 0.75) indicate high reliability, particularly in terms of inter-rater reliability and test-retest reliability. High ICC values were observed for the quantification of TRPV1 puncta (0.795), TRPV1 signal intensity (0.977), and determination of nerve fiber area (0.859). Thus, both MLAAF and DLAAP analyses show high reliability, indicating that both methods are suited to be used by different raters or across repeated testing conditions.

**Table 2:** Descriptive statistics of immunoreactivity TRPV1 quantity on nerve fibers.

Parameter	Manual analysis	MLAAF	DLAAP
Number of bioimages	28	28	28
Number of puncta (mean ± SD )	28.93 ± 24.29	24.04 ± 16.32	23.79 ± 19.76
TRPV1 MFI (AU) (mean ± SD )	–	134.06 ± 88.64	128.93 ± 93.93
Nerve fiber size (µm^2^) (mean ± SD )	–	230.63 ± 118.10	244.16 ± 162.88

MLAAF: Machine-learning-based automated image analysis in Fiji; DLAAP: Deep-learning-based automated image analysis in Python; AU: Arbitrary unit; MFI: Mean fluorescence intensity.

**Table 3:** Regression analysis of TRPV1 puncta on nerve fibers across methods.

Comparison	Spearmans’s correlation	*P* value (2-tailed)
MLAAF vs. manual	0.770	< 0.001
DLAAP vs. manual	0.751	< 0.001
MLAAF vs. DLAAP	0.711	< 0.001

MLAAF: Machine-learning-based automated image analysis in Fiji; DLAAP: Deep-learning-based automated image analysis in Python.

**Table 4:** Reliability analysis of TRPV1 intensity and nerve fiber size between MLAAF and DLAAP.

Parameter	Two-way mixed ICC	*P* value
TRPV1 Puncta	0.795	< 0.001
TRPV1 MFI (AU)	0.977	< 0.001
Nerve fiber size (µm^2^)	0.859	< 0.001

AU: Arbitrary unit; MFI: Mean fluorescence intensity; ICC: Intraclass correlation coefficient.

## Discussion

Neuropathic pain is a highly debilitating condition and not all patients can be treated satisfactorily. Targeting the TRPV1 receptor pharmacologically has shown some efficacy in clinical settings^18,19^, and individuals lacking the functional TRPV1 channel fail to respond to damaging stimuli^4^. Localizing TRPV1 on human tissue by immunofluorescence is challenging since there is not enough information on the specificity and sensitivity of the antibodies used to label human TRPV1^20,21^. Our study identified two commercially available antibodies (TRPV1-AL; TRPV1-NB, see material and methods) that are suited to label human TRPV1 in human skin. In contrast to the findings from a previous study^22^, TRPV1-AL labeled a subpopulation of small neurons in rat DRG. There are discrepant reports in the literature using different species and tissues regarding whether TRPV1-AL labels a 75 kDa band in Western Blot, which is 20 kDa smaller than the predicted molecular weight of TRPV1^23^, or a 95 kDa band, as expected^24^. TRPV1-NB was previously used to successfully stain human DRG, and its specificity was validated using pre-adsorption with a blocking peptide^25^. This was confirmed on human trigeminal ganglia, where it detected a 94 kDa TRPV1 band using Western blot^26^. We used the same antibody here and showed that it can also label TRPV1 in human skin with immunofluorescence labeling.

After immunofluorescence staining, manual counting of TRPV1-positive nerve fibers^13,27^ and visual assessments of fluorescent signals^28,29^ were the primary quantification approaches employed by researchers. However, although those studies showed quantification of TRPV1 immunosignals, an objective image analysis method is highly desirable. Manual, heuristic annotation by human experts can be expert-dependent, subjective, and can depend very much on signal-to-noise ratios^14^. This is especially critical when the rather ‘weak’ TRPV1 immunosignals are needed to group patient cohorts with the help of skin biopsies. Recent research has shown that bioimage analysis with machine learning or deep learning techniques can help to increase the objectivity and validity of immunosignal analysis in microscope images. For anti-TRPV1 analysis in human skin samples specifically, objective analysis of the staining needs to fulfill specific requirements. Important translational measures depend on reliable annotation of the nerve fibers, which is the basis for calculating the intraepidermal nerve fiber density (IENFD)^30–32^, determining nerve fiber branching points^32^, and quantifying clusters of nerve fibers crossing the basement membranes^31^. However, for detecting changes in TRPV1 expression on nerve fibers, reliable quantification parameters would help. Therefore, we established two automated analysis methods to provide researchers with more objective and efficient TRPV1 quantification options. Both computational methods, MLAAF and DLAAP, are best employed on bioimages with a segmentation of nerve fibers labelled with anti-PGP9.5. This segmentation guides the automated analysis of TRPV1-immunoreactive puncta, which then enables quantification of fluorescence intensities. MLAAF with the Weka trainable segmentation plugin in Fiji^33^ enabled automated image feature analysis without the need for GPU computing or extensive programming knowledge. DLAAP is based on a Python-based approach that uses deep learning algorithms and is especially useful for large-scale analysis of big datasets. Both machine learning and deep learning algorithms belong to the field of artificial intelligence^34^. These two automated methods are based on these two algorithms respectively to segment the nerve fibers (which is the most time-consuming task in the whole process) and use the same strategy for subsequent quantification.

Deep learning uses more hidden layers^35^ and tends to outperform machine learning models on large datasets with many complex features, but requires a higher computer configuration at runtime than traditional machine learning models^36^. These features enable deep learning to handle larger datasets^37^. In contrast, relatively simple machine learning is encoded as plug-ins that are installed in commonly used software such as Fiji, which do not require high configurations and can easily handle small data, making them more beginner-friendly. Depending on the requirements of the researcher and the amount of data, the researcher can choose the best method.

We acknowledge certain limitations that may impact the interpretation of our findings. Firstly, it is worthwhile to further test the specificity of antibodies against TRPV3 and TRPV4, which are highly expressed in human skin, in corresponding transfected HEK cells. Secondly, the experimental design may not be entirely intuitive for readers. Specifically, the low colocalization observed in the images of transfected HEK cells could be attributed to the use of two different vectors for TRPV1 and GFP expression. This approach may lead to variations in transfection efficiency, which can affect the degree of colocalization observed. Moreover, it needs to be considered that GFP accumulates in the cytosol, while TRPV1 is an ion channel that underlies ER translocation and membrane trafficking. Thirdly, all skin samples were collected from a single location above the ankle. This restricted sampling may not adequately represent the complexity of different skin regions which can exhibit varying anatomical and physiological characteristics.

In summary, our study provides a protocol for reliable labeling and quantification of TRPV1 in human skin samples.

## Methods

### Key resources table

**Table:** 

Reagent and dilution	Source	Identifier
TRPV1-SC Rat DRG: 1:100 Human skin: 1:100	Santa Cruz	Cat# sc-12498, RRID: AB_2241046
TRPV1-CM Rat DRG: 1:100 Human skin: 1:500	Chemicon	Cat# AB5889, RRID: AB_177543
TRPV1-AL Rat DRG: 1:500 HEK293 cells: 1:500 Human skin: 1:500	Alomone	Cat# ACC-030, RRID: AB_2313819
TRPV1-MK Rat DRG: 1:200 Human skin: 1:200	Millipore	Cat# AB5370, RRID: AB_2241031
TRPV1-NM Rat DRG: 1:500 Human skin: 1:500	Neuromics	Cat# GP14100, RRID: AB_1624142
TRPV1-NB Rat DRG: 1:100 HEK293 cells: 1:100 Human skin: 1:625	Novus Biologicals	Cat# NB120-3487, RRID: AB_788416
PGP9.5 HEK293 cells: 1:200 Human skin: 1:200	Bio-Rad Laboratories GmbH	Cat# 7863 − 2004, RRID: AB_620255
Sab-TRPV1-SC Rat DRG: 1:100 Human skin: 1:300	Jackson ImmunoResearch	Cat# 705-165-147, RRID: AB_2307351
Sab-TRPV1-CM Rat DRG: 1:100 Human skin: 1:300	Jackson ImmunoResearch	Cat# 711-165-152, RRID: AB_2307443
Sab-TRPV1-AL Rat DRG: 1:100 HEK293 cells: 1:200 Human skin: 1:200	Jackson ImmunoResearch	Cat# 711-165-152, RRID: AB_2307443
Sab-TRPV1-MK Rat DRG: 1:100 Human skin: 1:100	Jackson ImmunoResearch	Cat# 711-165-152, RRID: AB_2307443
Sab-TRPV1-NM Rat DRG: 1:100 Human skin: 1:200	Jackson ImmunoResearch	Cat# 106-165-003, RRID: AB_2337423
Sab-TRPV1-NB Rat DRG: 1:100 HEK293 cells: 1:200 Human skin: 1:200	Jackson ImmunoResearch	Cat# 711-165-152, RRID: AB_2307443
Sab-PGP9.5 HEK293 cells: 1:400 Human skin: 1:400	Jackson ImmunoResearch	Cat# 715-545-150, RRID: AB_2340846

### Rat dorsal root ganglion (DRG)

Animal protocols (Regierung von Unterfranken, Germany, #2-264) were approved by the animal care committee of the provincial government of Würzburg. All methods were performed according to the relevant guidelines and regulations by ARRIVE guidelines^38^. After euthanasia (detailed euthanasia steps in Supplement 1), DRG samples were obtained from 5-month-old Sprague Dawley rats (Charles River) at the L3-5 level. Cryosections of 10 μm thickness were air-dried and fixed with acetone (-20 °C) (Sigma-Aldrich).

### Human skin

The Ethics Committee of the Medical Faculty of the University of Würzburg approved the study (#98/20), and subjects were enrolled after written informed consent. This study was performed in accordance with relevant guidelines and followed the ethical guidelines of the Declaration of Helsinki. Human skin was taken from the distal leg 10 cm above the ankle with a biopsy punch under local anesthesia with lidocaine. The extracted skin samples were then fixed in fresh 4% buffered paraformaldehyde (PFH) for 30 min. Afterwards, the tissue was washed in 0.1 M pH 7.4 phosphate buffer and transferred to a 10% sucrose solution in phosphate buffer. The samples were snap-frozen in isopentane pre-cooled with liquid nitrogen on cork plates using Tissue-Tec^®^. Sections of 20 μm thickness were prepared with a cryostat.

### Transfected HEK293 cells

HEK293 cells were grown in DMEM with high glucose, GlutaMAX (Gibco, Cat# 35050061), 10% FCS, 100 units/ml penicillin, and 100 µg/ml streptomycin (Gibco, Cat# 15140-122). Coverslips (10 mm, Marienfeld) were placed in 4-well tissue culture dishes (Greiner, Cat# 176740) and coated with 0.1 mg/ml poly-L-lysine (PLL, Sigma-Aldrich). Cells were seeded on coverslips (200,000 cells/dish). For transfection, Lipofectamine 2000 (Invitrogen, Cat# 11668-019) was used at a ratio of 1 µg DNA per 2 µL Lipofectamine. TRPV1 was expressed with pcDNA3.1-hTRPV1 (a generous gift from Dr. Andreas Leffler, MHH Hannover, Germany)^10^. pCAGGS-GFP served as the transfection control. The medium was replaced after 24 h, and expression was maintained for 30–48 h.

### Method details

#### Workflow of antibody selection and validation

**Fig. 5:**
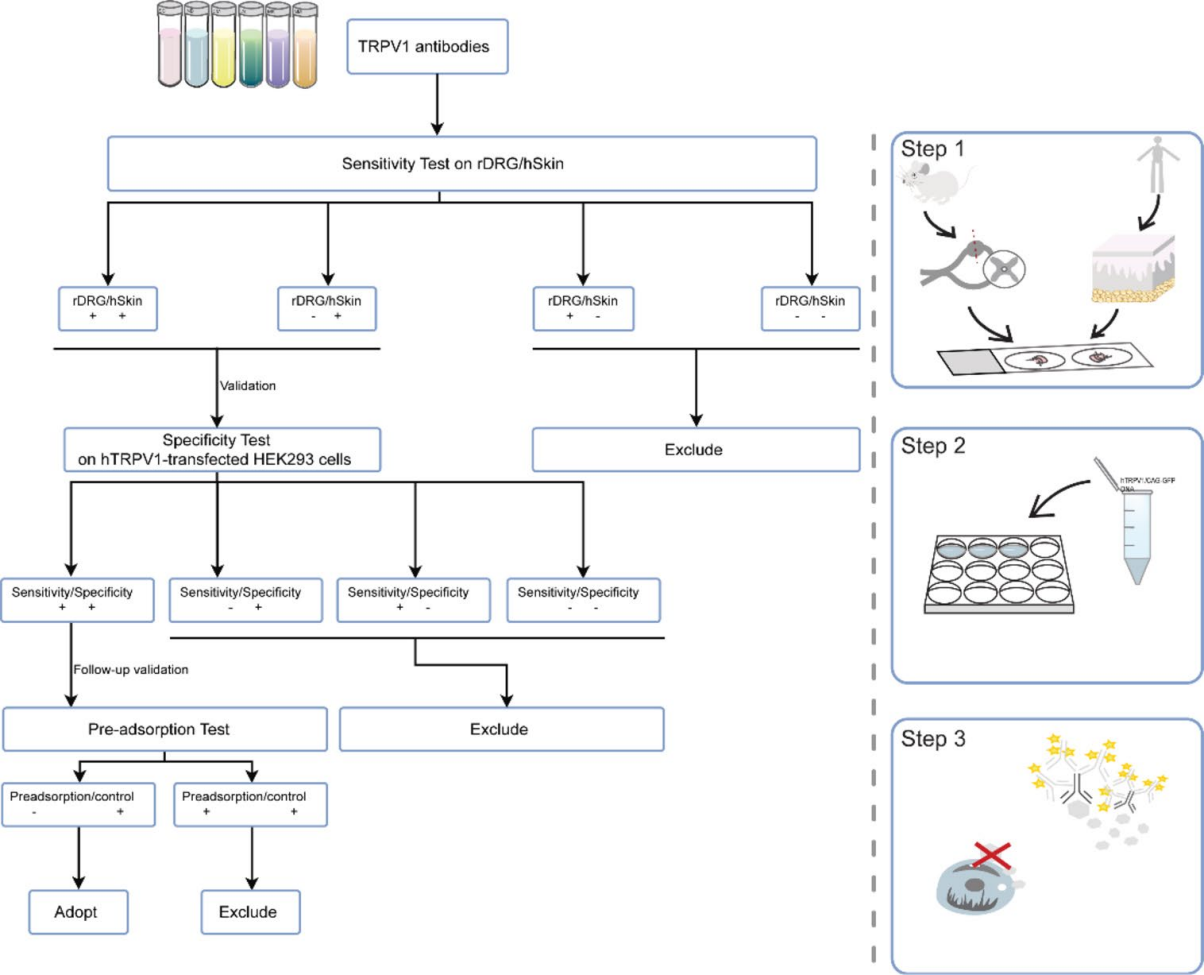
Workflow for selecting TRPV1 antibodies. Each TRPV1 antibody underwent a sensitivity assessment on rat dorsal root ganglion (rat DRG) and human skin samples. TRPV1 expression in rat DRG served as a reference before testing the performance of TRPV1 antibodies on human skin samples. The evaluation process consisted of three steps: Step 1 involved testing the antibody’s performance on both rat DRG and human skin. In Step 2, selected antibodies were analyzed with HEK293 cells expressing human-TRPV1. Finally, in Step 3, antibody performance was revalidated on human skin through a pre-adsorption test using a blocking peptide representing the TRPV1 antigen.

We selected commercially available antibodies against human TRPV1 that were recommended by the manufacturers or commonly used in published studies (see Table 1). Antibody performance was tested on rat DRG, human skin, and on recombinant human TRPV1. For revalidation, the antibody performance was evaluated after pre-adsorption with a corresponding blocking peptide (Fig. 5).

#### Immunofluorescence on rat DRG

DRG samples were dried on a heating plate. Sections were outlined with a PAP pen and washed in PBS. For blocking, we used a solution containing 10% BSA and 0.3% Triton™ X-100 (Sigma-Aldrich) for 2 h. For TRPV1 staining, primary anti-TRPV1 antibodies were applied in 1% BSA/PBS and incubated overnight at 4 °C in a humid chamber. After washing, the sections were incubated with the secondary antibody solution in 1% BSA/PBS for 1 h at room temperature (RT). The sections were embedded in an antifade mounting medium containing DAPI (Vector Laboratories), covered with coverslips, and then sealed with CoverGrip (Biotium Inc.).

#### Immunofluorescence on human skin biopsy

Human skin samples were air-dried at RT for 30 min. A hydrophobic barrier was created with a PAP pen. The sections were blocked by incubating them with 10% Bovine Serum Albumin/Phosphate-Buffered Saline for 30 min in a humid chamber. The primary antibodies (Key resources table) were prepared in a solution of 1% BSA/PBS with 0.3% Triton™ X-100 and were applied overnight at 4 °C, in a humid chamber. After washing three times with PBS, sections were incubated with secondary antibodies in 1% BSA/PBS for 2 h at RT. After washing with PBS, the samples were embedded in antifade mounting medium with DAPI and sealed with CoverGrip. Slides were stored at 4 °C.

#### Immunofluorescence on transfected HEK293 cells

Transfected HEK293 cells were fixed with 4% PFA for 20 min. After fixation, the cells were washed with PBS. Cells were permeabilized with 1% BSA/PBS and 1% Triton X-100 and blocked in a solution of 10% BSA/PBS for 1 h. After washing with 1% BSA/PBS, primary antibodies were used in 1% Triton™ X-100 and 2% BSA/PBS for 1 h. After washing three times with PBS, secondary antibodies were used in a solution of 1% Triton X-100 and 2% BSA/PBS for 1 h. The coverslips were again washed with PBS and labelled with DAPI (2 mg/ml stock solution, 1:10,000). Cells were embedded in Aqua-Polymount (Polysciences) and coverslips were sealed with CoverGrip.

#### Pre-adsorption of TRPV1 on human skin biopsy

For pre-adsorption, the TRPV1-AL and TRPV1-NB primary antibody solutions were mixed with their corresponding antigen solutions at a ratio of 10 µg peptide per 1 µg antibody. TRPV1-AL was pre-adsorbed with the blocking peptide from Alomone Labs (1:25, Cat# BLP-CC030, (C)EDAEVFKDSMVPGEK, Jerusalem, Israel). TRPV1-NB was pre-adsorbed with the blocking peptide from Invitrogen (1:31, Cat# PEP-202, T(7)DLGAAADPLQKDTC(21), Waltham, MA, USA). The mixture was gently mixed for 1 h at RT. Subsequently, the IF staining was carried out as described above.

#### Image acquisition

Bioimages for analysis were captured using a DMi8 Fluorescence microscope equipped with Lumencor’s bright engine and Thunder Imaging system (Leica Microsystems, Wetzlar, Germany). The images were acquired in 16-bit resolution with an HC PL FLUOTAR 40X objective and a resolution of 160 nm/pixel. Specifically, we utilized a 17 ms exposure time for all channels, with the fluorescence intensity modulation (FIM) light mode set at 30% intensity and no binning applied. Additionally, samples underwent z-stack acquisition with a step size of 1 μm over a total z-depth of 20 μm. During image acquisition, we applied deconvolution calculations (computational clearing methods) to enhance image quality.

For better presentation of details (Figs. 2 and 3), we utilized an inverted IX81 microscope equipped with an Olympus FV1000 confocal laser scanning system and a FVD10 SPD spectral detector. Confocal images (x, y, z) were acquired at 12-bit using an Olympus UPLSAPO 60X objective (N.A. 1.35) at ~ 100 nm/pixel. Quantification was performed on maximum intensity projection images.

#### Image processing

For objective quantification of the total numbers of TRPV1 immunoreactive signals located on nerve fibers, we employed three different methods: manual analysis, machine-learning (ML)-based automated image analysis in Fiji^33^ (Version: 2.9.0, https://fiji.sc/), and deep learning (DL)-based automated image analysis in Python (Version: 3.10.7, https://www.python.org/). These methods aimed to accurately quantify the TRPV1-immunopositive puncta along nerve fibers.

**Fig. 6:**
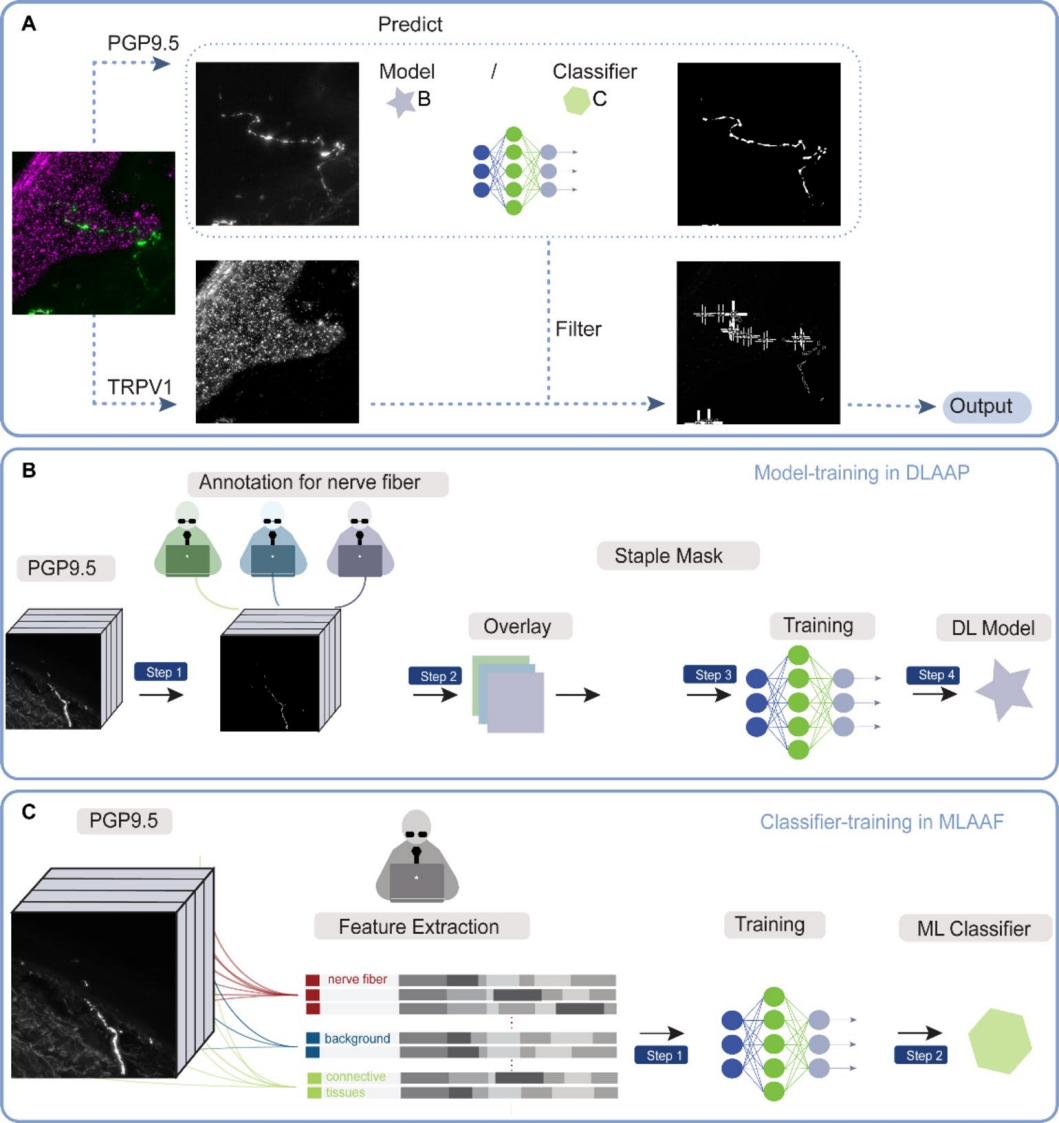
Workflow for automated quantification of TRPV1 immuno-positive puncta located on nerve fibers. (**A**) Deep-learning-based automated analysis in Python (DLAAP) and machine-learning-based automated analysis in Fiji (MLAAF) share a similar analysis concept. (**B**) Deep-learning models are based on annotations from three experts and were trained to predict nerve fibers for improved segmentation results. (**C**) Outline of the classifier training in MLAAF.

1. Manual counting analysis: 28 images of the region of interest were captured using a fluorescence microscope. Three independent observers manually counted the number of TRPV1-immunopositive puncta on PGP9.5-positive nerve fibers.
2. Deep-learning-based automated analysis in Python (DLAAP): The multi-channel images were split into individual channels using macros in Fiji. To segment the PGP9.5 channel images for nerve fibers, we employed deepflash2, a deep learning segmentation tool implemented in Python. Following guidelines for reproducible bioimage analysis^14,39^, we manually annotated 20 sample images by three experts. Subsequently, we overlaid each expert’s annotations as STAPLE masks. Then PGP9.5 images and STAPLE masks were employed to train the model. The Jaccard similarity coefficient score between the predicted masks and the annotated labels was used to assess model performance, also called intersection over union (IoU). The average dice score on three test images was 0.735. The IoU is defined as the size of the intersection divided by the size of the union of 2 sets of pixels (A, B): $\:\text{M}\text{I}\text{o}\text{U}(\text{A},\:\text{B}):=\frac{|\text{A}\cap\:\text{B}|}{|\text{A}\cup\:\text{B}|}$. The PGP9.5 model was then applied to segment all acquired PGP9.5 images. For the TRPV1 channel images, analysis was restricted to the predicted nerve fibers regions of the corresponding PGP9.5 images. We utilized the Big-FISH package^40^ to quantify TRPV1-immunopositive puncta with intensities exceeding the threshold value of 200 (Fig. 6A, B).
3. Machine-learning-based automated analysis in Fiji (MLAAF): The multi-channel images were split into individual channels using macros in Fiji. An expert manually classified 5 nerve fibers labeled with PGP9.5 images using the Trainable Weka segmentation plugin^41^, creating a classifier. The pre-trained classifier was then used to extract nerve fibers labeled with PGP9.5. The average dice score of IoU on three test images was 0.712. The resulting binary image representing the nerve fibers was converted into a mask, and the selected area was saved in the region of interest (ROI) manager. TRPV1-immunopositive puncta with intensities above a threshold value of 200 were counted using the “find maxima” detection function, specifically within the defined ROI (Fig. 6A, C).

### Statistics

All statistics were conducted using SPSS statistics (Version: 26, https://www.ibm.com/spss). Based on the descriptive characteristics of the data, including assessments of normality using the Shapiro-Wilk test and variance homogeneity using Levene’s test, the Kruskal-Wallis test and the Dunn post-hoc test were employed to identify group differences among the three methods. Spearman’s rank correlation coefficient was utilized to examine the correlation and correlation strength between pairs of methods. We also calculated the ICC (intraclass correlation coefficient) to assess the reliability and consistency of the measurement methods. The ICC was computed using a two-way mixed-effects model with absolute agreement, as this approach accounts for both systematic and random differences between the methods. The ICC is a statistical measure of rater agreement on the same subjects, with values less than 0.5, from 0.5 to 0.75, from 0.75 to 0.9, and greater than 0.90 considered as poor, moderate, good, and excellent agreement, respectively^42,43^.

## Electronic supplementary material

Below is the link to the electronic supplementary material.

Supplementary Material 1

## Data Availability

All original code, trained models (10.17632/4s6w7y4brx.2), and bioimages have been publicly deposited on Mendeley Data, available as of the publication date. Antibodies are provided in the Key Resources table.

## References

[1] Julius, D. TRP channels and pain. *Annu. Rev. Cell. Dev. Biol.* **29**, 355–384. 10.1146/annurev-cellbio-101011-155833 (2013). 24099085 10.1146/annurev-cellbio-101011-155833

[2] Caterina, M. J. et al. The capsaicin receptor: a heat-activated ion channel in the pain pathway. *Nature*. **389**, 816–824. 10.1038/39807 (1997). 9349813 10.1038/39807

[3] Middleton, S. J. et al. Studying human nociceptors: from fundamentals to clinic. *Brain*. **144**, 1312–1335. 10.1093/brain/awab048 (2021). 34128530 10.1093/brain/awab048 PMC8219361

[4] Katz, B. et al. Nociception and pain in humans lacking a functional TRPV1 channel. *J. Clin. Invest.* **133**10.1172/JCI153558 (2023). 10.1172/JCI153558 PMC9888381 36454632

[5] Sleczkowska, M. et al. Peripheral ion channel gene screening in painful- and painless-diabetic neuropathy. *Int. J. Mol. Sci.* **23**10.3390/ijms23137190 (2022). 10.3390/ijms23137190 PMC9266298 35806193

[6] Sleczkowska, M. et al. Peripheral ion channel genes screening in painful small fiber neuropathy. *Int. J. Mol. Sci.* **23**10.3390/ijms232214095 (2022). 10.3390/ijms232214095 PMC9696564 36430572

[7] Zhu, K. et al. Progress in the development of TRPV1 small-molecule antagonists: novel strategies for pain management. *Eur. J. Med. Chem.* **261**, 115806. 10.1016/j.ejmech.2023.115806 (2023). 37713804 10.1016/j.ejmech.2023.115806

[8] Derry, S., Rice, A. S., Cole, P., Tan, T. & Moore, R. A. Topical capsaicin (high concentration) for chronic neuropathic pain in adults. *Cochrane Database Syst. Rev.* **1**, CD007393. 10.1002/14651858.CD007393.pub4 (2017). 28085183 10.1002/14651858.CD007393.pub4 PMC6464756

[9] Xiao, T., Sun, M., Zhao, C. & Kang, J. TRPV1: a promising therapeutic target for skin aging and inflammatory skin diseases. *Front. Pharmacol.* **14**, 1037925. 10.3389/fphar.2023.1037925 (2023). 36874007 10.3389/fphar.2023.1037925 PMC9975512

[10] Erin, N. & Szallasi, A. Carcinogenesis and metastasis: focus on TRPV1-positive neurons and immune cells. *Biomolecules*. **13**10.3390/biom13060983 (2023). 10.3390/biom13060983 PMC10296534 37371563

[11] Denda, M. et al. Immunoreactivity of VR1 on epidermal keratinocyte of human skin. *Biochem. Biophys. Res. Commun.* **285**, 1250–1252. 10.1006/bbrc.2001.5299 (2001). 11478791 10.1006/bbrc.2001.5299

[12] Truini, A. et al. Joint European Academy of Neurology-European Pain Federation-Neuropathic Pain Special Interest Group of the International Association for the Study of Pain guidelines on neuropathic pain assessment. *Eur. J. Neurol.* **30**, 2177–2196. 10.1111/ene.15831 (2023). 37253688 10.1111/ene.15831

[13] Elitt, C. M. et al. Artemin overexpression in skin enhances expression of TRPV1 and TRPA1 in cutaneous sensory neurons and leads to behavioral sensitivity to heat and cold. *J. Neurosci.* **26**, 8578–8587. 10.1523/JNEUROSCI.2185-06.2006 (2006). 16914684 10.1523/JNEUROSCI.2185-06.2006 PMC6674358

[14] Segebarth, D. et al. On the objectivity, reliability, and validity of deep learning enabled bioimage analyses. *Elife*. **9**10.7554/eLife.59780 (2020). 10.7554/eLife.59780 PMC7710359 33074102

[15] Falk, M. et al. Heterochromatin drives compartmentalization of inverted and conventional nuclei. *Nature*. **570**, 395–399. 10.1038/s41586-019-1275-3 (2019). 31168090 10.1038/s41586-019-1275-3 PMC7206897

[16] Stirling, D. R. et al. CellProfiler 4: improvements in speed, utility and usability. *BMC Bioinform.* **22**, 433. 10.1186/s12859-021-04344-9 (2021). 10.1186/s12859-021-04344-9 PMC8431850 34507520

[17] Schwaid, A. G., Krasowka-Zoladek, A., Chi, A. & Cornella-Taracido, I. Comparison of the rat and human dorsal Root Ganglion Proteome. *Sci. Rep.* **8**, 13469. 10.1038/s41598-018-31189-9 (2018). 30194433 10.1038/s41598-018-31189-9 PMC6128859

[18] Arora, V., Campbell, J. N. & Chung, M. K. Fight fire with fire: neurobiology of capsaicin-induced analgesia for chronic pain. *Pharmacol. Ther.* **220**, 107743. 10.1016/j.pharmthera.2020.107743 (2021). 33181192 10.1016/j.pharmthera.2020.107743 PMC7969397

[19] Iftinca, M., Defaye, M. & Altier, C. TRPV1-targeted drugs in development for human pain conditions. *Drugs*. **81**, 7–27. 10.1007/s40265-020-01429-2 (2021). 33165872 10.1007/s40265-020-01429-2

[20] Cevikbas, F. et al. A sensory neuron-expressed IL-31 receptor mediates T helper cell-dependent itch: involvement of TRPV1 and TRPA1. *J. Allergy Clin. Immunol.* **133**, 448–460. 10.1016/j.jaci.2013.10.048 (2014). 24373353 10.1016/j.jaci.2013.10.048 PMC3960328

[21] Yang, X. L. et al. TRPV1 mediates astrocyte activation and interleukin-1beta release induced by hypoxic ischemia (HI). *J. Neuroinflammation*. **16**, 114. 10.1186/s12974-019-1487-3 (2019). 31142341 10.1186/s12974-019-1487-3 PMC6540554

[22] Toth, A. et al. Vanilloid receptor-1 (TRPV1) expression and function in the vasculature of the rat. *J. Histochem. Cytochem.* **62**, 129–144. 10.1369/0022155413513589 (2014). 24217926 10.1369/0022155413513589 PMC3902097

[23] Sand, C. A., Grant, A. D. & Nandi, M. Vascular expression of transient receptor potential vanilloid 1 (TRPV1). *J. Histochem. Cytochem.* **63**, 449–453. 10.1369/0022155415581014 (2015). 25809792 10.1369/0022155415581014 PMC4442824

[24] Chen, J. et al. Activation of TRPV1 channel by dietary capsaicin improves visceral fat remodeling through connexin43-mediated Ca2 + influx. *Cardiovasc. Diabetol.* **14**10.1186/s12933-015-0183-6 (2015). 10.1186/s12933-015-0183-6 PMC4340344 25849380

[25] Karai, L. et al. Deletion of vanilloid receptor 1-expressing primary afferent neurons for pain control. *J. Clin. Invest.* **113**, 1344–1352. 10.1172/JCI20449 (2004). 15124026 10.1172/JCI20449 PMC398431

[26] Pecze, L. et al. Human keratinocytes are vanilloid resistant. *PLoS One*. **3**, e3419. 10.1371/journal.pone.0003419 (2008). 18852901 10.1371/journal.pone.0003419 PMC2566593

[27] Sprague, J. M. et al. Bortezomib-induced neuropathy is in part mediated by the sensitization of TRPV1 channels. *Commun. Biol.* **6**10.1038/s42003-023-05624-1 (2023). 10.1038/s42003-023-05624-1 PMC10698173 38052846

[28] Pereira, M. P. et al. Application of an 8% capsaicin patch normalizes epidermal TRPV1 expression but not the decreased intraepidermal nerve fibre density in patients with brachioradial pruritus. *J. Eur. Acad. Dermatol. Venereol.* **32**, 1535–1541. 10.1111/jdv.14857 (2018). 29427475 10.1111/jdv.14857

[29] Weihrauch, T. et al. TRPV1 channel in human eosinophils: functional expression and inflammatory modulation. *Int. J. Mol. Sci.* **25**10.3390/ijms25031922 (2024). 10.3390/ijms25031922 PMC10856050 38339203

[30] Facer, P. et al. Differential expression of the capsaicin receptor TRPV1 and related novel receptors TRPV3, TRPV4 and TRPM8 in normal human tissues and changes in traumatic and diabetic neuropathy. *BMC Neurol.* **7**, 11. 10.1186/1471-2377-7-11 (2007). 17521436 10.1186/1471-2377-7-11 PMC1892784

[31] Gopinath, P. et al. Increased capsaicin receptor TRPV1 in skin nerve fibres and related vanilloid receptors TRPV3 and TRPV4 in keratinocytes in human breast pain. *BMC Womens Health*. **5**, 2. 10.1186/1472-6874-5-2 (2005). 15755319 10.1186/1472-6874-5-2 PMC554997

[32] Rage, M. et al. The time course of CO_2_ laser-evoked responses and of skin nerve fibre markers after topical capsaicin in human volunteers. *Clin. Neurophysiol.* **121**, 1256–1266. 10.1016/j.clinph.2010.02.159 (2010). 20347388 10.1016/j.clinph.2010.02.159

[33] Schindelin, J. et al. Fiji: an open-source platform for biological-image analysis. *Nat. Methods*. **9**, 676–682. 10.1038/nmeth.2019 (2012). 22743772 10.1038/nmeth.2019 PMC3855844

[34] Sarker, I. H., Furhad, M. H., Nowrozy, R. A. I. D. & Cybersecurity an overview, security intelligence modeling and research directions. *SN Comput. Sci.* **2**10.1007/s42979-021-00557-0 (2021).

[35] Aleesa, A. M., Zaidan, B. B., Zaidan, A. A. & Sahar, N. M. Review of intrusion detection systems based on deep learning techniques: coherent taxonomy, challenges, motivations, recommendations, substantial analysis and future directions. *Neural Comput. Appl.* **32**, 9827–9858. 10.1007/s00521-019-04557-3 (2020).

[36] Xin, Y. et al. Machine learning and deep learning methods for cybersecurity. *Ieee Access.* **6**, 35365–35381. 10.1109/Access.2018.2836950 (2018).

[37] LeCun, Y., Bengio, Y. & Hinton, G. Deep learning. *Nature*. **521**, 436–444. 10.1038/nature14539 (2015). 26017442 10.1038/nature14539

[38] Kilkenny, C., Browne, W. J., Cuthill, I. C., Emerson, M. & Altman, D. G. Improving bioscience research reporting: the ARRIVE guidelines for reporting animal research. *PLoS Biol.* **8**, e1000412. 10.1371/journal.pbio.1000412 (2010). 20613859 10.1371/journal.pbio.1000412 PMC2893951

[39] Griebel, M. et al. Deep learning-enabled segmentation of ambiguous bioimages with deepflash2. *Nat. Commun.* **14**, 1679. 10.1038/s41467-023-36960-9 (2023). 36973256 10.1038/s41467-023-36960-9 PMC10043282

[40] Imbert, A. et al. FISH-quant v2: a scalable and modular tool for smFISH image analysis. *RNA*. **28**, 786–795. 10.1261/rna.079073.121 (2022). 35347070 10.1261/rna.079073.121 PMC9074904

[41] Arganda-Carreras, I. et al. Trainable Weka Segmentation: a machine learning tool for microscopy pixel classification. *Bioinformatics*. **33**, 2424–2426. 10.1093/bioinformatics/btx180 (2017). 28369169 10.1093/bioinformatics/btx180

[42] Shrout, P. E. & Fleiss, J. L. Intraclass correlations - uses in assessing rater reliability. *Psychol. Bull.* **86**, 420–428. 10.1037/0033-2909.86.2.420 (1979). 18839484 10.1037//0033-2909.86.2.420

[43] Sun, L. et al. Deep learning quantification of percent steatosis in donor liver biopsy frozen sections. *EBioMedicine*. **60**, 103029. 10.1016/j.ebiom.2020.103029 (2020). 32980688 10.1016/j.ebiom.2020.103029 PMC7522765

