# Supplementary material for: Antibody selection and automated quantification of TRPV1 immunofluorescence on human skin
Source: Sci Rep. 2024 Nov 18;14:28496. doi: 10.1038/s41598-024-79271-9 (PMC11574049; doi:10.1038/s41598-024-79271-9)
Supplement: Supplementary file 1 — Supplementary Material 1 [file 41598_2024_79271_MOESM1_ESM.docx]

Supplement for euthanasia

To begin the euthanasia procedure, ensure that the euthanasia chamber is clean and transparent for optimal visibility. The chamber is equipped with a piece of paper at the bottom. When the gas flows in, care is taken to ensure that no perceptible noise is generated. Connect the CO2 gas cylinder to the pressure-reducing regulator and flow meter. Set the flow rate of CO2 to 50% of the chamber volume per minute. Gently place the rat inside the euthanasia chamber, ensuring it has enough space to move comfortably without overcrowding. Once the rat is positioned, activate the CO2 flow by turning on the switch on the regulator to release gas into the chamber. Continuously observe the rat during this process for signs of unconsciousness, such as recumbency and absence of movement, which typically occur within 3 minutes after initiating CO2 flow. It is crucial to maintain CO2 flow for at least 1 minute after respiratory arrest is observed to ensure death. After confirming respiratory arrest, utilize a decapitation method as a confirmatory step to ensure death.
